# Latent profile analysis of emergency nurses’ pre-triage competence and its associated factors: a multicenter cross-sectional study

**DOI:** 10.3389/fmed.2026.1899998

**Published:** 2026-07-27

**Authors:** Qin Huang, Shifang Mao, Lei Yue, Bo Liu, Ying Wang, Guorong Cai, Fang Yang, Shanbing Hou

**Affiliations:** 1 Deyang People’s Hospital, Deyang, Sichuan, China; 2 Affiliated Hospital of Southwest Medical University, Luzhou, Sichuan, China; 3 Sichuan Provincial People’s Hospital, Chengdu, Sichuan, China; 4 The First People’s Hospital of Longquanyi District Chengdu, Chengdu, Sichuan, China; 5 Mianyang Central Hospital, Mianyang, Sichuan, China; 6 Suining Central Hospital, Suining, Sichuan, China; 7 Department of Nursing, The First Affiliated Hospital of USTC, Division of Life Sciences and Medicine, University of Science and Technology of China, Hefei, Anhui, China

**Keywords:** China, clinical decision-making, emergency nursing, latent profile analysis, nursing informatics, pre-triage competence, professional development

## Abstract

**Background:**

Pre-triage competence is a critical component of emergency nursing that directly influences patient prioritization, clinical safety, and departmental efficiency. However, few studies have explored the heterogeneity of this competence among emergency nurses or the factors shaping different competence profiles. This study aimed to identify distinct latent subgroups of pre-triage competence among emergency nurses and to examine demographic, educational, and professional factors associated with competence levels.

**Methods:**

A multicenter, cross-sectional survey was conducted from July 1 to August 1, 2025, across 12 secondary and tertiary hospitals in Sichuan Province, China. A total of 367 emergency nurses completed a structured, self-administered questionnaire comprising the Emergency Pre-Triage Competence Scale, Nursing Process Application Ability Scale, and Nursing Informatics Competency Scale. Latent profile analysis (LPA) was performed in Mplus 8.3 to identify potential subgroups of pre-triage competence, followed by multinomial logistic regression to determine predictors of class membership.

**Results:**

Model fit indices supported a three-class solution representing low (21.8%), moderate (30.8%), and high (47.4%) levels of pre-triage competence. Nurses in the high-competence group demonstrated consistently higher mean scores across clinical synthesis and illness-severity judgment, communication and coordination, and comprehensive emergency collaboration domains. Univariate analyses revealed significant between-class differences in professional title, years of service, educational level, hospital grade, monthly income, and specialty certification (all *p* < 0.001). Multivariate regression identified higher professional title, longer emergency department experience, bachelor’s degree or above, employment in tertiary hospitals, higher monthly income, critical/emergency specialty certification, and stronger nursing process and informatics competencies as independent predictors of belonging to moderate- or high-competence groups.

**Conclusion:**

Emergency nurses’ pre-triage competence is heterogeneous and can be classified into three distinct profiles. Higher educational attainment, extended professional experience, higher rank, and stronger process and informatics abilities are key determinants of superior competence. These findings underscore the importance of targeted training and professional development strategies to enhance pre-triage performance and ensure patient safety in emergency care settings.

## 1 Introduction

Worldwide, emergency departments (EDs) are facing the dual challenges of a steadily increasing patient volume and strained medical resources, as well as the need to ensure the timeliness and safety of care delivery. As the first step in the emergency care process, triage plays a crucial role in ensuring patient safety and improving the efficiency of ED operations by rapidly assessing the severity of patients’ conditions and allocating medical resources appropriately (1). Numerous studies have shown that high-quality triage not only shortens patient waiting times and alleviates ED overcrowding but also reduces adverse events caused by triage delays or misjudgments, thereby improving the efficiency of care for critically ill patients (2–4).

In recent years, with the shift of emergency nursing models toward competency-based professional practice and the rapid development of AI-assisted triage, digital health technologies, and information management systems, the role of emergency nurses in triage has gradually expanded from traditional patient classification to a more comprehensive function integrating rapid clinical assessment, risk identification, evidence-based decision-making, interprofessional collaboration, application of digital tools, and resource coordination (5, 6). Recent studies and systematic reviews indicate that although digital transformation has improved the efficiency and standardization of emergency triage, nurses’ professional judgment remains the core determinant of triage quality, with digital technologies serving primarily as decision-support tools rather than substitutes for clinical judgment (7). Consequently, how to comprehensively assess and enhance emergency nurses’ triage competence has become a major focus in the field of emergency nursing.

Nursing competence is typically defined as the integrated application of knowledge, skills, clinical judgment, professional values, and professional attitudes in specific contexts (8). In the emergency department setting, nurses’ triage competence is not only reflected in the accurate prioritization of patient conditions but also encompasses multidimensional abilities such as communication and coordination, teamwork, risk prediction, and emergency response (9). Although triage processes have been continuously optimized in recent years, existing studies still report that nurses’ triage accuracy ranges from approximately 60 to 82%, with significant variations observed across regions, institutions, and levels of clinical experience (10). These findings suggest that even under standardized triage criteria, substantial individual differences in emergency nurses’ triage competence persist, and the underlying characteristics of these differences warrant further investigation.

Furthermore, previous studies have indicated that emergency nurses’ triage competence is influenced by multiple factors, including educational background, clinical experience, continuing education, organizational support, work environment, and digital literacy (11–14). However, current evaluations of triage competence primarily rely on total score comparisons or traditional regression models, with limited attention paid to the latent heterogeneity inherent in nursing competence as a multidimensional construct. In-depth exploration of latent subgroups of nurses with different competence profiles and their associated factors remains insufficient. Moreover, with the continued advancement of digital emergency nursing, digital literacy has increasingly become a critical factor influencing clinical decision-making and triage quality; however, related evidence remains limited.

Latent Profile Analysis (LPA) is a person-centered statistical approach used to identify unobserved subgroups based on multiple continuous indicators. Compared with traditional variable-centered methods, LPA is more effective in revealing the internal heterogeneity of nursing competence and distinguishing distinct competence profiles (15). Previous studies have suggested that factors such as clinical experience, educational level, specialty certification, workload, and organizational support may influence emergency nurses’ triage competence. However, evidence regarding latent classification patterns of triage competence and their associated determinants remains scarce.

Therefore, this study adopted a multicenter cross-sectional design and employed Latent Profile Analysis to identify latent subgroups of emergency nurses’ triage competence based on three dimensions: clinical synthesis and condition assessment, communication and coordination, and comprehensive emergency collaboration. Furthermore, we examined the effects of demographic characteristics, educational background, occupational factors, nursing process application skills, and nursing informatics proficiency on latent class membership. This study aims to provide a theoretical basis for stratified management, targeted training, and workforce allocation of emergency nurses, and to support continuous improvement in the quality of emergency nursing care.

## 2 Methods

### 2.1 Study design

This study adopted a multicenter, cross-sectional design to systematically evaluate the current status and heterogeneity of emergency nurses’ pre-triage competence in Sichuan Province, China. A structured, self-administered questionnaire survey was conducted using the Questionnaire Star online platform. Latent profile analysis (LPA) was performed to classify nurses into subgroups according to three key domains of pre-triage competence—clinical synthesis and illness-severity judgment, communication and coordination, and comprehensive emergency collaboration—followed by multinomial logistic regression to identify demographic, educational, and professional factors associated with class membership. This design enabled a quantitative exploration of individual differences in triage competence while minimizing recall bias and enhancing representativeness across hospitals of different accreditation levels.

### 2.2 Setting

The survey was implemented in the emergency departments of 12 comprehensive hospitals (secondary and tertiary) distributed across Sichuan Province, China. All participating hospitals provide 24-h emergency services and maintain multidisciplinary teams routinely responsible for pre-triage and initial patient assessment. The data collection period lasted from July 1 to August 1, 2025, during which eligible emergency nurses were invited to complete the online questionnaire anonymously through the secure Questionnaire Star platform.

### 2.3 Participants

Eligibility criteria. Inclusion criteria were: (1) registered nurses holding a valid nursing qualification certificate; (2) currently working on the front line in an emergency department with ≥ 1 year of emergency clinical experience; and (3) provided informed consent and volunteered to participate. Exclusion criteria were: (1) off duty during the survey window (e.g., on leave); and (2) visiting/rotating nurses.

### 2.4 Variables

Primary outcome. Latent classes of pre-triage competence derived from three observed domains of the Emergency Pre-Triage Competence Scale: (1) clinical synthesis and illness-severity judgment, (2) communication and coordination, and (3) comprehensive medical emergency response and collaboration. Exposures/predictors and potential confounders. Demographics and job characteristics (age, sex, marital status, job title, position, years in emergency department, employment type, monthly income, hospital level, highest/first academic degree), specialty qualification status (critical/emergency specialty nurse certificate), nursing process application ability, and nursing informatics competency. No diagnostic criteria were applicable.

### 2.5 Data sources/measurement

General information. A study team–developed form captured demographic and job-related variables (age, sex, education, job title, position, hospital level, marital status, years in emergency department, monthly income, employment type, and specialty certificate status).

Emergency Pre-Triage Competence Scale. Originally developed by Bijani et al. (16) and adapted into Chinese by Hou et al. (17). The localization process followed the Brislin translation model, incorporating translation-back-translation and cultural adaptation, and resulted in a Chinese version of the scale following a pilot study. The Chinese version of the scale consists of 35 items covering three dimensions: clinical integration and condition assessment, communication and coordination, and comprehensive emergency collaboration. It uses a 5-point Likert scale (1 = Strongly Disagree, 5 = Strongly Agree). Reliability and validity tests showed that the Cronbach’s *α* for the total scale was 0.987, split-half reliability was 0.911, and test–retest reliability was 0.932; Regarding content validity, the scale-level content validity index (S-CVI) was 0.968, and the item-level content validity index (I-CVI) ranged from 0.778 to 1.000; regarding construct validity, exploratory factor analysis identified three common factors, accounting for 79.182% of the total variance. These results indicate that the Chinese version of the scale possesses good reliability and validity.

Nursing Process Application Ability Scale. Developed by Koy et al. (18), and sinicized by Wang et al. (19), assessed on a 5-point Likert scale with higher scores indicating stronger application ability; Cronbach’s *α* in this sample was 0.987.

Nursing Informatics Competency Scale. Developed by Luo Hong et al. (20), 32 items/5 dimensions, 5-point Likert (1 = very inconsistent to 5 = very consistent); higher scores indicate stronger informatics competency; Cronbach’s *α* was 0.957. All instruments were administered online with standardized instructions.

### 2.6 Bias

**Figure 1:**
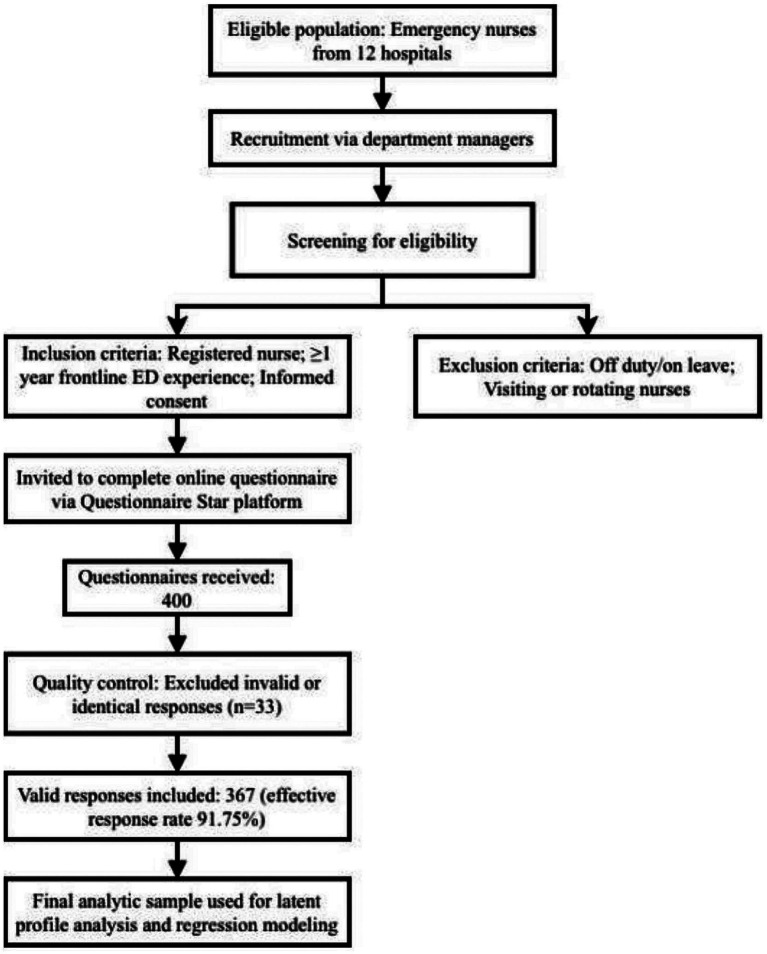
The overall recruitment and inclusion process.

Recruitment was conducted using convenience sampling. The research team communicated in advance with the heads of nursing and the heads of the emergency departments at each participating hospital to obtain a list of nurses who met the inclusion criteria and their work schedules for the past month. The survey was conducted from July 1 to August 1, 2025. Designated liaisons from each department (typically the head nurse or a designated teaching secretary) explained the study’s purpose, anonymity, and the right to voluntarily withdraw to eligible nurses on duty during two time slots: after the morning shift handover and after the end of the night shift. Subsequently, links or QR codes for the QuestionnaireStar platform were distributed uniformly through the department’s WeChat work group. To cover nurses with different work schedules, the survey included staff rotating through day, evening, and night shifts. Nurses who were on leave or off-duty on the day of the survey were not contacted for follow-up or asked to complete the questionnaire later. A total of 400 questionnaires were distributed to eligible nurses, and 400 were returned. After quality control, 367 valid responses were obtained, resulting in a response rate of 91.75%. The recruitment and screening process for study participants is shown in Figure 1.

### 2.7 Study size

Following multivariable analysis convention, the target sample size was 5–10 participants per predictor. With 35 candidate predictors and anticipating 20% invalid questionnaires, the required sample was 210–420. We collected 400 responses and analyzed 367 valid cases, meeting the prespecified range.

### 2.8 Quantitative variables

Scale scores were treated as continuous variables (higher values reflect stronger ability for the nursing process and informatics scales). Categorical predictors (e.g., job title, hospital level, income) were coded into ordered categories as specified below for regression modeling.

### 2.9 Statistical methods

All statistical analyses were performed using Mplus 8.3 and SPSS 27.0. Descriptive analyses were first conducted to summarize sample characteristics: continuous variables approximately following a normal distribution were expressed as mean ± standard deviation (SD), and categorical variables were presented as frequency and percentage. Differences among latent classes of pre-triage competence were initially examined using chi-square tests for categorical variables. To identify distinct patterns of emergency nurses’ pre-triage competence, latent profile analysis (LPA) was performed in Mplus 8.3 based on three observed domain scores—clinical synthesis and illness-severity judgment, communication and coordination, and comprehensive emergency collaboration. First, conduct a comprehensive comparison of AIC, BIC, and adjusted BIC, giving priority to models with lower information criteria values while paying attention to the rate of decline. If the rate of decline in each criterion slows significantly after adding a category, this supports a more concise model. Second, require an entropy value of ≥0.80 as the standard for acceptable classification quality; Third, both the LMRT and BLRT must reach statistical significance (*p* < 0.05), indicating that the K-class model is significantly superior to the K-1-class model; finally, a comprehensive assessment is conducted based on practical significance and category interpretability—the proportion of samples in each category must be no less than 5% of the total sample, and the scoring patterns across each dimension must demonstrate clinical discriminative power. In the event of inconsistencies among statistical indicators, the BLRT results are given priority, and the final decision is made in conjunction with clinical interpretability. After determining the best-fitting latent class solution, a multinomial (unordered) logistic regression model was constructed in SPSS 27.0 to examine associations between latent class membership (using the low competence class as the reference category) and a set of potential predictors including job title, years of emergency department experience, highest education level, hospital accreditation level, monthly income, critical/emergency specialty nurse certification status, nursing process application ability, and nursing informatics competency. Variables with *p* < 0.05 in univariable analyses were included in the multivariable model to adjust for confounding effects. Categorical predictors were coded as follows: job title (nurse = 1, nurse practitioner = 2, charge nurse = 3, associate senior or above = 4); years in emergency department (≤5 = 1, 6–10 = 2, 11–20 = 3, ≥21 = 4); highest education (junior college or below = 0, bachelor’s degree or above = 1); hospital level (secondary or below = 0, tertiary = 1); monthly income (≤5,000 = 1, 5,001–8,000 = 2, 8,001–10,000 = 3, ≥10,001 = 4); and critical/emergency specialty nurse (no = 0, yes = 1). Nursing process application ability and nursing informatics competency were treated as continuous variables based on their original scale scores.

The Questionnaire Star platform required completion of key items before submission; therefore, missing data were minimal. Responses failing preset quality-control criteria (e.g., unrealistically short completion time or identical answers) were excluded *a priori*, and no data imputation was conducted, resulting in a complete-case analysis. As a sensitivity analysis, alternative LPA models with one to four classes were compared using multiple fit indices (AIC, BIC, adjusted BIC, entropy) and likelihood-ratio tests (LMRT and BLRT) to confirm the robustness of class enumeration. All statistical tests were two-sided, and *p* < 0.05 was considered statistically significant.

### 2.10 Ethical considerations

All participants signed informed consent forms before joining the study. The research received ethical approval from the Ethics Committee of the First Affiliated Hospital of the University of Science and Technology of China (2024-ky394).

## 3 Results

### 3.1 Model fit indices for latent profile analysis

**Figure 2:**
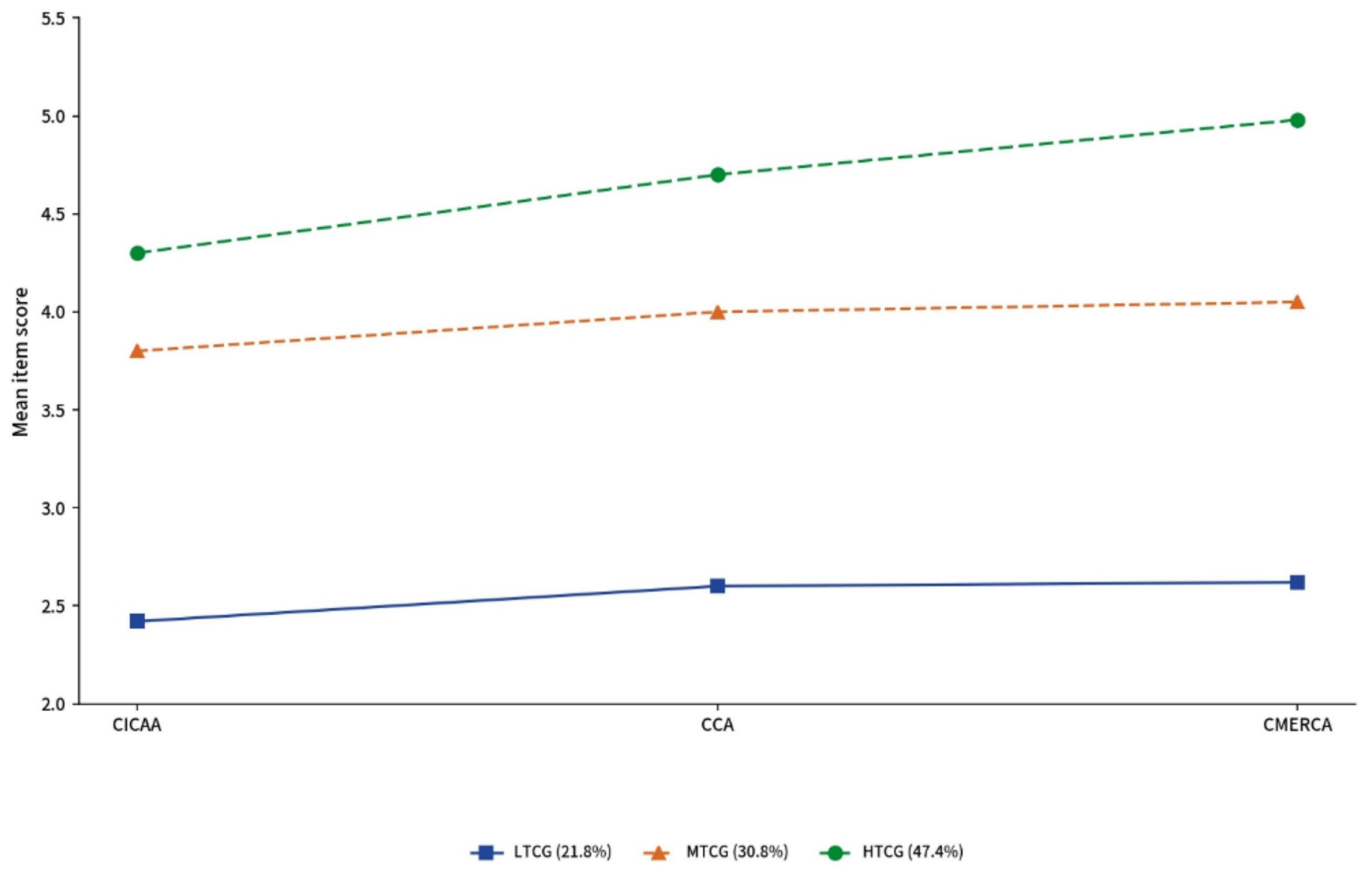
Model for latent profile analysis.

As shown in Table 1, model fit indices were compared across one- to four-class latent profile solutions to determine the optimal classification of emergency nurses’ pre-triage competence. The Akaike Information Criterion (AIC), Bayesian Information Criterion (BIC), and adjusted BIC (aBIC) values decreased progressively as the number of classes increased from one to four, suggesting improved model fit with additional classes. The entropy values for all models exceeded 0.98, indicating excellent classification accuracy. However, while both the Lo–Mendell–Rubin likelihood ratio test (LMRT) and bootstrap likelihood ratio test (BLRT) were significant (*p* < 0.05) when comparing the two- and three-class models, the LMRT became non-significant (*p* = 0.109) for the four-class model, suggesting that the addition of a fourth class did not yield a significantly better fit. Consequently, the three-class model was selected as the optimal and most parsimonious solution, with class membership probabilities of 21.8, 30.8, and 47.4%, respectively. As illustrated in Figure 2, these three latent classes displayed distinct mean score profiles across the three competence domains—clinical synthesis and illness-severity judgment (CICAA), communication and coordination ability (CCA), and comprehensive medical emergency response and collaboration ability (CMERCA). The high-competence group (HTCG) consistently showed the highest mean item scores across all domains, followed by the moderate-competence group (MTCG), whereas the low-competence group (LTCG) exhibited uniformly lower scores. This progressive gradient in domain performance provides strong support for the discriminative validity and interpretability of the three-class model.

**Table 1:** Model fit indices for latent profile analysis of emergency nurses’ pre-triage competence (*n* = 367).

Number of classes	AIC	BIC	aBIC	Entropy	LMRT (*p*)	BLRT (*p*)	Class probability (%)
1	2895.633	2919.065	2900.029	—	—	—	—
2	1921.303	1960.356	1928.630	0.984	0.000	0.000	23.4/76.6
3	1651.814	1706.489	1662.072	0.983	0.043	0.000	21.8/30.8/47.4
4	948.411	1018.707	961.600	0.998	0.109	0.000	7.9/48.0/30.2/13.9

AIC, Akaike Information Criterion; BIC, Bayesian Information Criterion; aBIC, adjusted Bayesian Information Criterion; LMRT, Lo–Mendell–Rubin likelihood ratio test; BLRT, bootstrap likelihood ratio test. Smaller AIC, BIC, and aBIC values indicate better model fit; higher entropy (closer to 1) indicates better classification accuracy. A *p* < 0.05 for LMRT or BLRT indicates that the K-class model fits significantly better than the (K–1)-class model.

### 3.2 Demographic and occupational characteristics of participants

The demographic and occupational characteristics of the 367 emergency nurses included in this study are summarized in Table 2. The majority of participants were female (85.3%), and more than half were aged 25–34 years (55.3%), followed by those aged 35–44 years (32.7%). Most nurses held the professional titles of senior nurse (42.0%) or charge nurse (41.4%), while only a small proportion (6.0%) had attained an associate senior nurse or higher qualification. In terms of years of service in the emergency department (ED), approximately one-third (37.1%) had 11–20 years of experience, 27.2% had 6–10 years, and 25.6% had ≤ 5 years. Regarding educational background, 68.1% of nurses had a junior college degree or below as their first qualification, whereas 81.5% held a bachelor’s degree or above as their highest degree. The vast majority of participants worked in tertiary hospitals (87.5%), with only 12.5% employed in secondary or lower-level institutions. Most nurses were contract-based employees (89.6%), and 85.3% held staff nurse positions, while smaller proportions served as team leaders (10.4%) or head nurses (4.4%). In terms of monthly income, about half of the respondents (51.0%) earned 5,001–8,000 RMB, while 21.8% earned ≤ 5,000 RMB, and 27.3% reported incomes above 8,000 RMB. The majority were married (71.9%), whereas 24.8% were unmarried and 3.3% were divorced or widowed. Additionally, 43.6% of nurses were certified critical/emergency specialty nurses, indicating substantial representation of professionally trained personnel within the sample.

**Table 2:** Demographic and occupational characteristics of emergency nurses (*n* = 367).

Variable	Category	*n*	%
Sex	Male	54	14.7
	Female	313	85.3
Age (years)	< 25	14	3.8
	25–34	203	55.3
	35–44	120	32.7
	≥ 45	30	8.2
Professional title	Nurse	39	10.6
	Senior nurse	154	42.0
	Charge nurse	152	41.4
	Associate senior nurse or above	22	6.0
Years of service in emergency department	≤ 5	94	25.6
	6–10	100	27.2
	11–20	136	37.1
	≥ 21	37	10.1
First academic degree	Junior college or below	250	68.1
	Bachelor’s degree or above	117	31.9
Highest academic degree	Junior college or below	68	18.5
	Bachelor’s degree or above	299	81.5
Hospital level	Secondary or below	46	12.5
	Tertiary	321	87.5
Employment status	Permanent	38	10.4
	Contract-based	329	89.6
Current position	Staff nurse	313	85.3
	Team leader	38	10.4
	Head nurse	16	4.4
Monthly income (RMB)	≤ 5,000	80	21.8
	5,001–8,000	187	51.0
	8,001–10,000	59	16.1
	≥ 10,001	41	11.2
Marital status	Unmarried	91	24.8
	Married	264	71.9
	Divorced/Widowed	12	3.3
Certified critical/emergency specialty nurse	No	207	56.4
	Yes	160	43.6

Data are presented as number (percentage). RMB, Chinese Yuan (Renminbi).

### 3.3 Univariate analysis of latent classes of pre-triage competence

As shown in Table 3, significant differences were observed across the three latent classes of emergency nurses’ pre-triage competence in terms of professional title, years of service in the emergency department (ED), highest academic degree, hospital level, monthly income, and certified critical/emergency specialty nurse status (all *p* < 0.001). Nurses in the low-competence group were predominantly junior in rank and experience, with 36.3% holding the title of nurse and over half (53.8%) having ≤5 years of ED service. In contrast, the high-competence group comprised a greater proportion of senior professionals, including 49.4% charge nurses and 9.2% associate senior nurses or above, with more than half (54.0%) having 11–20 years of experience and 16.1% exceeding 21 years. A clear educational gradient was also evident: 94.8% of nurses in the high-competence group held a bachelor’s degree or higher, compared with only 46.3% in the low-competence group. Similarly, 97.7% of nurses in the high-competence group worked in tertiary hospitals, whereas one-third (33.8%) of those in the low-competence group were employed in secondary or lower-level hospitals. Income levels were positively associated with pre-triage competence—more than 40% of high-competence nurses earned above 8,000 RMB, while half (51.2%) of low-competence nurses earned ≤5,000 RMB. Furthermore, holding a critical/emergency specialty certification was markedly more prevalent among nurses in the moderate- and high-competence groups (51.3 and 54.6%, respectively) than in the low-competence group (8.8%). These findings suggest that higher educational attainment, longer ED experience, employment in tertiary hospitals, greater professional rank, and specialty certification are all associated with higher levels of pre-triage competence among emergency nurses.

**Table 3:** Univariate analysis of demographic and occupational variables by latent classes of pre-triage competence among emergency nurses [*n* (%)].

Variable	Low competence group (*n* = 80)	Moderate competence group (*n* = 113)	High competence group (*n* = 174)	*χ* ^2^	*p*
Professional title				**86.791**	**< 0.001**
Nurse	29 (36.3)	10 (8.8)	0 (0.0)		
Senior nurse	34 (42.5)	48 (42.5)	72 (41.4)		
Charge nurse	17 (21.3)	49 (43.4)	86 (49.4)		
Associate senior nurse or above	0 (0.0)	6 (5.3)	16 (9.2)		
Years of service in emergency department				**99.410**	**< 0.001**
≤ 5	43 (53.8)	33 (29.2)	18 (10.3)		
6–10	32 (40.0)	34 (30.1)	34 (19.5)		
11–20	5 (6.3)	37 (32.7)	94 (54.0)		
≥ 21	0 (0.0)	9 (8.0)	28 (16.1)		
Highest academic degree				**87.735**	**< 0.001**
Junior college or below	43 (53.8)	16 (14.2)	9 (5.2)		
Bachelor’s degree or above	37 (46.3)	97 (85.8)	165 (94.8)		
Hospital level				**49.530**	**< 0.001**
Secondary or below	27 (33.8)	15 (13.3)	4 (2.3)		
Tertiary	53 (66.3)	98 (86.7)	170 (97.7)		
Monthly income (RMB)				**85.270**	**< 0.001**
≤ 5,000	41 (51.2)	24 (21.2)	15 (8.6)		
5,001–8,000	39 (48.8)	61 (54.0)	87 (50.0)		
8,001–10,000	0 (0.0)	21 (18.6)	38 (21.8)		
≥ 10,001	0 (0.0)	7 (6.2)	34 (19.5)		
Certified critical/emergency specialty nurse				**50.815**	**< 0.001**
No	73 (91.3)	55 (48.7)	79 (45.4)		
Yes	7 (8.8)	58 (51.3)	95 (54.6)		

Data are presented as number (percentage). *χ*^2^ = chi-square statistic. *p* < 0.05 was considered statistically significant.

### 3.4 Multivariate analysis of factors associated with latent classes of pre-triage competence

Results of the multinomial logistic regression analysis are presented in Table 4. After adjusting for significant variables identified in the univariate analyses, several demographic and professional characteristics remained independently associated with class membership, using the low-competence group as the reference category. For the moderate-competence group, higher professional title (OR = 1.880, 95% CI: 1.037–3.408, *p* = 0.037), higher academic degree (OR = 3.095, 95% CI: 1.274–7.517, *p* = 0.013), higher monthly income (OR = 1.914, 95% CI: 1.039–3.525, p = 0.037), certification as a critical/emergency specialty nurse (OR = 4.772, 95% CI: 1.804–12.624, *p* = 0.002), and greater scores in both nursing process application ability (OR = 1.039, 95% CI: 1.010–1.068, *p* = 0.007) and nursing informatics competency (OR = 1.022, 95% CI: 1.005–1.039, *p* = 0.012) were significantly associated with belonging to this class. For the high-competence group, multiple predictors showed even stronger associations. Nurses with longer ED experience (OR = 1.867, 95% CI: 1.033–3.375, *p* = 0.039), higher professional title (OR = 2.099, 95% CI: 1.061–4.152, *p* = 0.033), a bachelor’s degree or above (OR = 6.352, 95% CI: 1.927–20.937, *p* = 0.002), and employment in tertiary hospitals (OR = 6.956, 95% CI: 1.561–30.991, *p* = 0.011) were more likely to be categorized in the high-competence group. In addition, higher monthly income (OR = 2.036, 95% CI: 1.067–3.885, *p* = 0.031), critical/emergency specialty certification (OR = 2.980, 95% CI: 1.046–8.493, *p* = 0.041), stronger nursing process application ability (OR = 1.143, 95% CI: 1.085–1.203, *p* < 0.001), and higher nursing informatics competency (OR = 1.051, 95% CI: 1.024–1.078, *p* < 0.001) were independent predictors of higher pre-triage competence. Collectively, these results indicate that advanced education, extended clinical experience, employment in tertiary hospitals, higher professional rank, and stronger nursing process and informatics skills substantially increase the likelihood of belonging to moderate- or high-competence groups among emergency nurses.

**Table 4:** Multinomial logistic regression analysis of factors associated with latent classes of pre-triage competence among emergency nurses (*n* = 367).

Variable	Moderate competence group			High competence group		
	OR	95% CI	*p*	OR	95% CI	*p*
Professional title	1.880	1.037–3.408	0.037	2.099	1.061–4.152	0.033
Years of service in ED	1.140	0.664–1.958	0.635	1.867	1.033–3.375	0.039
Highest academic degree	3.095	1.274–7.517	0.013	6.352	1.927–20.937	0.002
Hospital level	1.105	0.400–3.052	0.848	6.956	1.561–30.991	0.011
Monthly income (RMB)	1.914	1.039–3.525	0.037	2.036	1.067–3.885	0.031
Certified critical/emergency specialty nurse	4.772	1.804–12.624	0.002	2.980	1.046–8.493	0.041
Nursing process application ability	1.039	1.010–1.068	0.007	1.143	1.085–1.203	< 0.001
Nursing informatics competency	1.022	1.005–1.039	0.012	1.051	1.024–1.078	< 0.001

OR, odds ratio; CI, Confidence interval; ED, Emergency department. The reference category was the low pre-triage competence group, and a two-tailed *p* < 0.05 was considered statistically significant.

## 4 Discussion

The core findings of this study indicate that emergency nurses’ triage competence does not follow a single linear distribution but instead exhibits latent categorical characteristics with marked structural differences. From an individual-centered perspective, these results further support the hypothesis of heterogeneity in nursing competence—namely, that in complex, high-workload, and time-sensitive clinical settings, nurses’ competence is more likely to manifest as stable configurational patterns rather than continuous variation along a single dimension. This finding suggests that triage competence may have an intrinsic hierarchical structure rather than a homogeneous distribution assumed in traditional research. Meanwhile, emergency nursing practice is gradually shifting from a task-oriented model toward a multidimensional competency framework centered on clinical decision-making, risk identification, and interprofessional collaboration. Against this backdrop, the present study identifies competency subtypes from a person-centered perspective, which helps to more precisely characterize differences in the competency structures of emergency nurses and provides a theoretical basis for precision management based on competency profiles.

This study found that nurses with higher professional titles, longer experience in emergency care, higher educational attainment, employment in tertiary hospitals, higher income levels, and specialized nursing certifications were more likely to belong to the medium-to-high triage competency classes. These findings are generally consistent with previous studies and (21, 22), based on a multicenter sample, further support the cumulative nature of competency development among emergency nurses. Specifically, the accumulation of clinical experience may enhance pattern recognition and risk assessment abilities in complex and uncertain environments, thereby improving consistency in patient triage and prioritization (13). At the same time, nurses with higher educational attainment typically demonstrate stronger critical thinking and evidence-based practice capabilities, which may be associated with higher levels of clinical reasoning in dynamic care settings (23). Tertiary hospitals, characterized by higher case complexity, heavier workloads, and more developed multidisciplinary collaboration systems, provide nurses with intensive clinical exposure and learning opportunities for competency development; these organizational features may facilitate competency enhancement (14). Furthermore, specialized nursing training emphasizes standardized assessment processes and simulation-based training, which are associated with improved performance in clinical workflow management and emergency response. This finding is consistent with previous evidence indicating that specialized training enhances the efficiency and safety of emergency patient management (13). Recent international reviews have also highlighted that increasing uncertainty in emergency nursing practice in the post-pandemic era has further amplified the importance of experience accumulation and structured training in shaping competency differences, while simultaneously driving the evolution of nursing competency frameworks toward multidimensional integration (7).

This study further found a significant association between nursing process application competency and nursing informatics competency, and higher levels of triage competence. This finding suggests that emergency nurses’ competence depends not only on traditional clinical skills but is also closely linked to structured clinical reasoning and digital competencies. The ability to apply the nursing process (assessment–diagnosis–planning–implementation–evaluation) reflects a systematic clinical reasoning framework. In high-workload emergency settings characterized by incomplete and rapidly changing information, this competency may enable nurses to integrate fragmented clinical data more efficiently and is associated with greater consistency in triage decision-making (24).

Furthermore, the role of nursing informatics competency is particularly prominent in the context of ongoing digital transformation in healthcare. With the increasing adoption of electronic health records, clinical decision support systems, real-time monitoring systems, and AI-assisted triage tools in emergency settings, nurses are required to process heterogeneous data from multiple sources and translate them into clinically meaningful insights (25). Consequently, nursing informatics competency has evolved from basic system operation skills to a more comprehensive capability set, including information integration, data interpretation, digital communication, and information security awareness. Recent studies suggest that AI-assisted triage systems may improve the efficiency of risk identification and optimize resource allocation; however, they remain decision-support tools rather than substitutes for clinical judgment (26). At the same time, algorithmic outputs may introduce a degree of bias, potentially affecting nurses’ independent clinical judgment. Therefore, within a human–machine collaborative decision-making model, nursing informatics competency encompasses not only technical operational skills but also the ability to critically evaluate and appropriately contextualize algorithmic outputs. In digital emergency nursing practice, nursing informatics competency and clinical judgment are therefore complementary. The findings of this study support the value of human–machine collaborative decision-making in emergency triage, indicating that the development of high-level triage competence relies on both accumulated clinical experience and advanced digital health literacy.

With the continued advancement of digital health technologies and artificial intelligence in emergency care, the competency structure of emergency nurses is undergoing systematic transformation. In the future, triage competence will extend beyond traditional clinical judgment to include digital health literacy, proficiency in AI-based tools, and clinical informatics skills (27). Accordingly, hospital administrators may consider implementing stratified staffing and targeted training strategies based on competency profiles, while gradually integrating AI-assisted systems and electronic decision-support tools into triage workflows to improve efficiency and consistency. For nurses in the low-competence group, training should focus on strengthening fundamental clinical assessment skills and standardized triage procedures, combined with mentorship and simulation-based training to enhance risk identification and decision consistency. For nurses in the medium-competence group, progression toward higher competence may be facilitated through advanced scenario-based training and the development of interprofessional collaboration skills. Nurses in the high-competence group can be assigned more complex triage responsibilities and play key roles in guidance and quality control within the team. At the same time, it is essential to maintain the central role of clinical judgment in the application of digital tools. Through a collaborative “technology support plus professional judgment” model, the complementary strengths of humans and machines can be leveraged to improve emergency department efficiency and triage consistency while ensuring patient safety.

## 5 Strengths and limitations

Strengths include a multicenter design spanning secondary and tertiary hospitals; the use of LPA to capture heterogeneity at the person-level rather than assuming homogeneity; and prespecified, theory-informed predictors covering education, role seniority, organizational context, and proximal skills.

This study has several important limitations that warrant careful interpretation. First, the cross-sectional design limits causal inference between individual and institutional factors and pre-triage competence. It is plausible that nurses with higher competence preferentially seek employment in tertiary hospitals, which in turn provide richer learning environments that further reinforce competence.

Second, this study employed convenience sampling and was limited to Sichuan Province, which imposes clear limitations on its external generalizability. First, there are geographical limitations: the disease spectrum, patient volume, and level of informatization in triage for emergency department patients in Sichuan Province may differ from those in other provinces; therefore, the profile categories and their composition ratios should not be directly extrapolated to other regions. Second, there is recruitment bias: questionnaires were distributed by department managers during breaks between shifts. Nurses on duty during peak periods were less likely to respond, which may have resulted in underrepresentation of groups with high burnout or low self-efficacy, thereby affecting the estimated associations between relevant factors and profile categories. Nevertheless, the distribution of age and professional titles in the sample closely aligns with the overall characteristics of the province’s emergency nursing workforce, and the results remain valuable for reference in similar comprehensive hospitals in central and western China. Future studies should include institutions across multiple provinces and at various administrative levels to verify the stability of these profiles.

Third, since the primary variables are all self-reported data—even though validated scales were used and strict quality control measures were implemented—two types of bias still exist: First, social expectation bias, whereby nurses may tend to overestimate their own abilities to conform to professional expectations. This systematic upward bias may narrow the intervals between profile categories, thereby weakening the discriminant validity between the high-ability and moderate-ability groups; Second, an asymmetric deviation between self-assessment and objective ability—where low-ability individuals tend to overestimate their abilities while high-ability individuals tend to underestimate them—may lead to the misclassification of individuals in borderline categories, thereby distorting the direction or intensity of the effects of relevant factors. Nevertheless, the self-assessment method remains widely accepted in large-scale, multicenter surveys where objective performance metrics are unavailable. In the future, objective indicators such as triage accuracy, wait times, and adverse event rates should be incorporated and supplemented with peer or supervisor evaluations to perform multi-source calibration, thereby verifying the discriminant stability of the profile classifications.

Fourth, residual confounding cannot be excluded despite multivariable adjustment; unmeasured institutional factors (e.g., ED occupancy, staffing ratios, protocol standardization, or simulation-based training) may simultaneously affect both competence and its predictors. Fifth, the latent profile model assumed local independence and continuous normal distributions for scale data; deviations from these assumptions could subtly distort class boundaries, and class membership probabilities were not corrected for potential misclassification in subsequent regressions. Furthermore, clustering by hospital was not accounted for, meaning that shared environmental effects may have reduced standard errors. Lastly, the study did not explore cross-level interactions, such as whether education moderates the impact of hospital level or income on competence. Future multilevel or longitudinal designs linking individual, team, and institutional characteristics to objective performance metrics are recommended to validate and extend these findings.

Prospective, multi-region studies should evaluate whether movement between latent classes is achievable through targeted interventions (e.g., specialty certification, informatics upskilling, simulation-based training) and whether such transitions translate into measurable improvements in patient-centered and operational outcomes (safety events, left-without-being-seen, ED length of stay) under varying crowding conditions. Mixed-methods designs could unpack how organizational culture and protocol fidelity shape the enactment of competence at the front door, while implementation studies could test pragmatic bundles that integrate staffing algorithms, audit-feedback, and digital decision support at pre-triage.

## 6 Conclusion

In summary, pre-triage competence among emergency nurses is heterogeneous and naturally clusters into three ordered profiles. Higher education, seniority, tertiary-hospital setting, specialty certification, and stronger nursing process and informatics competencies independently characterize nurses in the higher-competence profiles. Health systems seeking to improve safety and flow at the ED front end should adopt competency-based deployment and development strategies that target these leverage points while strengthening organizational supports for triage practice.

## Data Availability

The original contributions presented in the study are included in the article/supplementary material, further inquiries can be directed to the corresponding author/s.
